# Associations of blood pressure components with risks of cardiovascular events and all‐cause death in a Chinese population: A Prospective Study

**DOI:** 10.1111/jch.14529

**Published:** 2022-06-24

**Authors:** Zhongying Zhang, Xiang Gu, Zhe Tang, Shaochen Guan, Hongjun Liu, Xiaoguang Wu, Yan Zhao, Xianghua Fang

**Affiliations:** ^1^ Geriatric department Xuanwu Hospital Capital Medical University Beijing China; ^2^ Evidence‐based Medical Center Xuanwu Hospital Capital Medical University Beijing China; ^3^ Medical affair department Beijing Friendship Hospital, Capital Medical University Beijing China; ^4^ Beijing Geriatric Healthcare Center Xuanwu Hospital Capital Medical University Beijing China; ^5^ Education department Xuanwu Hospital Capital Medical University Beijing China

**Keywords:** cardiovascular events, cohort study, hypertension, pulse pressure

## Abstract

The associations of blood pressure components with cardiovascular risks and death remain unclear, and the definition of wide pulse pressure (PP) is still controversial. Using data from 1257 participants without a history of cardiovascular disease, who were followed for 4.84 years, we performed multivariable Cox regression analyses to assess how systolic blood pressure (SBP), diastolic blood pressure (DBP), and PP contribute to risks of cardiovascular events and all‐cause death. Among all participants, SBP and PP were significantly associated with the risks of cardiovascular events and all‐cause death (all *p* < .05). DBP was not significantly associated with the risk of all‐cause death; rather, it was only associated with a marginally significant 1% increased risk for cardiovascular events (*p* = 0.051). In participants aged < 65 years, DBP was significantly associated with a 3% increased risk for cardiovascular events (hazard ratio [HR]: 1.03, 95% confidence interval [95% CI]: 1.01–1.06). The association between PP and cardiovascular events appeared to be J‐shaped in comparison to participants with the lowest‐risk PP (50–60 mmHg), with adjusted HRs of 1.71 (95% CI: 1.03–2.85), 1.63 (95% CI: 1.00–2.68), and 2.13 (95% CI: 1.32–3.43) in the <50, 60.0–72.5, and ≥72.5 mmHg subgroups, respectively. The optimal cutoff points of a wide PP for predicting the risks of cardiovascular events and all‐cause death were 70.25 and 76.25 mmHg, respectively. SBP and PP had a greater effect on cardiovascular risk, whereas DBP independently influenced cardiovascular events in middle‐aged participants. Considerable PP alterations should be avoided in antihypertensive treatment.

## 1 INTRODUCTION

Hypertension remains one of the most important contributors to cardiovascular events and deaths worldwide.^1^ Robust evidence from clinical trials has shown the benefit of antihypertensive medication in reducing adverse health outcomes in patients with hypertension.^2^, ^3^, ^4^ However, previous studies investigating the effects of blood pressure components (systolic blood pressure [SBP], diastolic blood pressure [DBP], and pulse pressure [PP]) on cardiovascular risks and death provided equivocal results.^5^, ^6^ Some studies reported that SBP levels could be used to better predict cardiovascular events compared to other blood pressure components;^6^, ^7^ in contrast, other studies reported that DBP levels showed a J‐curve^8^, ^9^ or were not associated with the prediction of cardiovascular events.^10^, ^11^ PP remains a poorly studied parameter receiving less attention in clinical trials.^12^ Current hypertension guidelines do not include a target PP,^13^, ^14^, ^15^ and the definition of wide PP remains controversial, ^16^, ^17^, ^18^ which may subject patients to an independent risk factor, leading to poor outcomes.

Equivocal results of different blood pressure components on cardiovascular risks and death might be explained by age differences. Middle‐aged individuals commonly have a higher DBP in the early stage of hypertension. With aging and progressing arterial stiffening, older people are prone to having a higher SBP, a lower DBP, and a wide PP. Previous studies found that the relative importance of blood pressure components to coronary heart disease^19^ and stroke^20^ risks changed with aging in Framingham Heart Study and the MOnica, Risk, Genetics, Archiving, and Monograph Project. However, the question of which blood pressure components predict cardiovascular events across age ranges has not been fully evaluated in the Chinese population.

Therefore, this study aimed to explore the associations of blood pressure components with cardiovascular events and all‐cause death in different age groups. We performed a prospective cohort study using data from the Beijing Longitudinal Study of Aging (BLSA), a longitudinal study on middle‐aged and older community‐dwelling individuals in Beijing, China. The present study findings may have clinical health implications for the prevention of cardiovascular events in middle‐aged and elderly people.

## 2 METHODS

The data of the current study were collected from the BLSA, which was designed to investigate the health status and cardiovascular risk factors of the Chinese middle‐aged and elderly population. Details on the design and implementation of the BLSA whose data were used in this study have been reported previously.^21^, ^22^, ^23^ Briefly, Beijing consists of 18 administrative districts that are classified into the following three categories according to their degree of urbanization and economic status: eight main cities, five suburbs, and five extended suburbs in 1992. First, the following three districts were selected by stratification–random–clustering sampling from each category: Xuanwu District (urban), Daxing County (suburb), and Huairou County (extended suburb). Secondly, specific communities/villages were randomly selected from these districts based on demographic characteristics and the educational level of the population. Thirdly, all residents in sampled communities/villages were invited to participate in baseline interviews, questionnaire surveys, physical examinations, and laboratory tests. Similar surveys were conducted every 3–4 years with seven waves of follow‐up data since 1992. The current study employed data from the last two waves. The baseline data of the current study were based on BLSA findings of the 2009 survey, and follow‐up data were obtained from the 2014/2015 survey.

Individuals who lived in one of the sampled communities/villages and who could communicate normally and complete the questionnaire were enrolled in this study in 2009. The exclusion criteria were as follows: (1) individuals with a history of myocardial infarction or stroke, (2) individuals with severe congestive heart failure (CHF) (New York Heart Association classes III–IV), (3) individuals who refused to participate in blood pressure measurements or to provide blood samples, and (4) individuals who refused to sign the informed consent form. In total, 2468 participants were enrolled. Among them, 164 participants were excluded because of a history of myocardial infarction, stroke, or severe CHF. Furthermore, 1010 of 2304 participants withdrew because they refused to provide blood samples, and 37 participants were excluded because important information was missing. Finally, 1257 participants were enrolled, and their data were carefully collected. The cohort was followed up from November 2014 to February 2015.

The Ethics Committee of Xuanwu Hospital, Capital Medical University, Beijing, China, approved this study (No.2018‐038). This study was performed in accordance with the Declaration of Helsinki, and all participants provided written informed consent.

### 2.1 Baseline data collection

Well‐trained investigators conducted face‐to‐face interviews and questionnaire surveys to collect baseline information from June 2009 to August 2009. Participants were asked to answer questions to the best of their knowledge. The standardized questionnaire included questions on socioeconomic conditions and demographics (name, age, sex, date of birth, and marital status), health‐related behaviors (smoking habit and alcohol consumption), medical history (hypertension, diabetes mellitus, coronary heart disease, and stroke), and medications (antihypertensive therapy, lipid‐lowering therapy, and hypoglycemic therapy). Physical examinations were performed by investigators at the participant's home or designated sites. Using a digital blood pressure monitor (Omron HEM‐4021; Omron, Kyoto, Japan), blood pressure was measured according to a standard protocol in a sitting position on the non‐dominant arm after a 5‐min rest. The mean value of two measurements within 2 min was used for the analysis.

Blood samples were collected on the morning following the completion of the questionnaire. After 12 h fasting, blood samples were collected into a 3.5 ml Greiner Bio‐One Z Serum Sep Clot Activator tube and were centrifuged within 1 h at 3000 rpm for 15 min. Separated serum samples were stored in a refrigerator at 2–8°C until testing. All laboratory measurements were performed by routine methods or as per the user manual of the test kits in a commercial laboratory (IPE Center for Clinical Laboratory, Beijing, China) within 24 h.

### 2.2 Definitions and laboratory examinations

PP was calculated as the difference between SBP and DBP. Wide PP was defined as either ≥60^24^ or ≥100 mmHg.^12^ Hypertension was defined as an SBP of ≥140 mmHg and/or a DBP of ≥90 mmHg and/or use of antihypertensive medication in the past 2 weeks.^25^ Dyslipidemia was defined according to current lipid levels or use of anti‐dyslipidemia medication within 2 weeks. Cutoff values were defined as any of the following: a total cholesterol level of ≥6.22 mmol/L, triglycerides level of ≥2.26 mmol/L, a high‐density lipoprotein cholesterol level of <1.04 mmol/L, or a low‐density lipoprotein cholesterol level of ≥4.14 mmol/L.^26^ Furthermore, diabetes mellitus was defined by a fasting plasma glucose level of ≥7.0 mmol/L, the use of antidiabetic medication, or a history of diabetes mellitus.^26^ Smoking and drinking were subcategorized as former, current, and never. Former smokers/drinkers were those who had a history of smoking/drinking and had stopped for at least 2 years; current smokers/drinkers were defined as those who were still smoking/drinking frequently or occasionally till the time of the investigation, and participants with no history of smoking/drinking were identified as never smokers/drinkers. In this report, we defined smokers/drinkers as former or current smokers/drinkers.^21^

High‐sensitivity C‐reactive protein (hs‐CRP) levels were measured using a high‐sensitivity nephelometric assay with the Behring Nephelometer II system (Dade Behring, Marburg, Germany). Fasting glucose levels were determined using the glucose oxidase–peroxidase assay. High‐density and low‐density lipoprotein cholesterol levels were measured using the direct assay. Total cholesterol, triglycerides, and creatinine levels were determined with the standard enzymatic method using the Hitachi Clinical Analyzer (Hitachi 7600, Hitachi, Tokyo, Japan).

The estimated glomerular filtration rate (eGFR) was calculated according to the abbreviated Modification of Diet in Renal Disease formula as follows:^27^ eGFR (in ml/min/1.73 m^2^) = 175 × serum creatinine^− 1.154^ × age ^− 0.203^ × 0.742 [if female].

### 2.3 Prospective follow‐ups and endpoints

Follow‐up surveys for an average of 4.84 years were performed to confirm the endpoints for each participant enrolled in the baseline survey. New cases of cardiovascular events and deaths during the follow‐up period were collected. A standardized questionnaire was used to investigate cardiovascular events and included following questions: “Have you been told by a doctor that you have been diagnosed with myocardial infarction or stroke since 2009?” or “Have you had a percutaneous coronary intervention, coronary artery bypass surgery, or peripheral vascular surgery since 2009?” If the answer to any of the questions is yes, well‐trained investigators proceeded to collect relevant information as follows: diagnosis time, hospital where diagnosis was conducted, electrocardiogram, myocardial enzyme records, brain computed tomography, brain magnetic resonance imaging, detailed medical history of the diagnosis, hospital discharge records, other imaging data, and whether the disease was diagnosed by a neurologist or cardiologist. Other available information, such as personal health files and inpatient medical records, was also obtained from local community hospitals in the three districts, Xuanwu, and Friendship Hospitals. The medical diagnostic team of two attending physicians from the Department of Geriatrics of Xuanwu Hospital and Friendship Hospital assessed cardiovascular events.

Death records were obtained from the medical records, Center for Disease Control and Prevention, and relatives of participants. Causes of death were codified according to the principles of the 10th version of the International Classification of Diseases.

In this study, cardiovascular events were defined as a composite of coronary events (i.e., fatal, or non‐fatal myocardial infarction, percutaneous coronary intervention, coronary artery bypass surgery, or sudden cardiac death), stroke events (i.e., fatal or non‐fatal stroke and ischemic or hemorrhagic stroke), and peripheral vascular events (i.e., peripheral vascular surgery).^21^

### 2.4 Statistical analyses

Baseline characteristics were summarized according to different age groups (<65 years vs. ≥65 years) before a primary outcome. Normally distributed continuous variables are expressed as the mean ± standard deviations. Differences between groups were assessed using independent samples *t*‐tests. Non‐normally distributed continuous data are expressed as median values (interquartile ranges), and median differences between groups were assessed using the Kruskal–Wallis test. Categorical variables are reported as numbers (percentages), and Pearson's chi‐squared test was used to determine the differences between categorical variables.

A multivariate Cox regression model was used to evaluate the contribution of SBP (continuous variable), DBP (continuous variable), PP (continuous variable), and wide PP (categorical variable) to the risks of cardiovascular events and all‐cause death in all participants and those belonging to the abovementioned age groups. For each blood pressure parameter, we also categorized the study participants into quartiles as follows: SBP <125 (reference), 125–137, 137–150.5, and ≥150.5 mmHg; DBP <69.5, 69.5–76.5 (reference), 76.5–83.5, and ≥83.5 mmHg; and PP <50, 50–60 (reference), 60–72.5, and ≥72.5 mmHg. The Cox regression model comprised the following scenarios: crude (unadjusted), model 1 (adjusted for sex and age), and model 2 (adjusted for variables in model 1 plus smoking status, alcohol consumption, diabetes mellitus, dyslipidemia, hs‐CRP, eGFR, and antihypertensive treatment). Unless specified, all results are given for the fully adjusted model. The hazard ratios (HRs) and 95% confidence intervals (95% CIs) of cardiovascular events and all‐cause death were calculated using the Cox regression model.

Using receiver operating characteristic (ROC) analysis, the area under the curve (AUC) of the ROC curve was calculated to evaluate the effects of SBP, DBP, and PP on the risks of cardiovascular events and all‐cause death in all participants and those belonging to the two age groups. Additionally, the optimal cutoff values of the levels of PP for the prediction of cardiovascular events and all‐cause death were determined by ROC curves, applying Youden's index.^28^

All reported *p*‐values are two‐sided. Statistical significance was set at *p <* .05, and statistical analyses were performed using SPSS 19.0 (SPSS Inc., IBM, Armonk, NY, USA) and Prism 8 (GraphPad Software, San Diego, CA, USA).

## 3 RESULTS

### 3.1 Participants’ baseline information

Baseline characteristics of the participants in the entire study population (1257 participants) and in both age subgroups are shown in **Table** **1**. The mean age of all participants was 69.16 ± 8.10 years, and 550 (43.76%) were men. The hypertension prevalence in participants aged ≥65 years versus those <65 years was 66.76% (528/791) versus 50.21% (234/466; *p <* .001). The mean PP was 62.25 ± 17.20 mmHg. The SBP levels, PP levels, and proportions of individuals with wide PP (either ≥60 or ≥100 mmHg) were significantly higher in older participants than in younger participants (all *p <* .05). Conversely, the DBP levels were significantly higher in younger participants (*p <* .001). Additionally, the hsCRP, total cholesterol, and triglycerides levels were significantly higher in older participants than in younger participants (all *p <* .05), whereas the proportion of smokers was significantly lower in older participants (*p* = .012).

**TABLE 1:** Baseline characteristics of the participants by age

Variables	Overall (n = 1257)	Participants <65 years of age (n = 466)	Participants ≧65 years (n = 791)	*p*‐value
Age (years)	69.16 ± 8.10	60.51 ± 2.95	74.26 ± 5.37	<.001
Male	550 (43.76%)	194 (41.63%)	356 (45.01%)	.244
Smoking	386 (30.71%)	163 (34.98%)	223 (28.19%)	.012
Drinking	386 (30.71%)	158 (33.91%)	228 (28.82%)	.059
Diabetes	218 (17.34%)	69 (14.81%)	149 (18.84%)	.068
Hypertension	762 (60.62%)	234 (50.21%)	528 (66.76%)	<.001
Antihypertensive therapy in hypertensive patients	391 (51.31%)	116 (49.57%)	275 (52.01%)	.440
Dyslipidemia	736 (58.55%)	302 (64.81%)	434 (54.87%)	.001
Systolic blood pressure, mmHg	138.93 ± 20.35	134.98 ± 18.61	141.29 ± 20.98	<.001
Diastolic blood pressure, mmHg	76.68 ± 10.93	78.45 ± 10.21	75.63 ± 11.22	<.001
Pulse pressure, mmHg	62.25 ± 17.20	56.53 ± 14.88	65.66 ± 17.59	<.001
Wide pulse pressure (≥60 mmHg)	628 (49.96%)	169 (36.27%)	459 (58.03%)	<.001
Wide pulse pressure (≥100 mmHg)	46 (3.66%)	7 (1.50%)	39 (4.93%)	.002
Total cholesterol, mmol/L	5.89 ± 1.15	6.01 ± 1.13	5.81 ± 1.16	.002
Triglycerides, mmol/L	1.31 (0.97‐1.88)^a^	1.38 (1.02‐2.07)^a^	1.27 (0.94‐1.79)^a^	.001
HDL‐C, mmol/L	1.23 ± 0.27	1.22 ± 0.28	1.23 ± 0.27	.625
LDL‐C, mmol/L	2.96 ± 0.75	2.92 ± 0.74	2.98 ± 0.75	.144
hsCRP, mg/L	0.03 (0.01‐0.17)^a^	0.03 (0.01‐0.14)^a^	0.04 (0.01‐0.18)^a^	.010
eGFR, ml/min per 1.73 m^2^	58.80 ± 15.37	59.84 ± 11.87	58.18 ± 17.08	.064

*Notes*: Data are presented as means ± SD or n (%).

**Abbreviations**: HDL‐C, high‐density lipoprotein cholesterol‐C; LDL‐C, low‐density lipoprotein cholesterol‐C; hsCRP, high‐sensitivity C‐reactive protein; eGFR, estimated glomerular filtration rate.

^a^ Presented as median (interquartile range).

**FIGURE 1:**
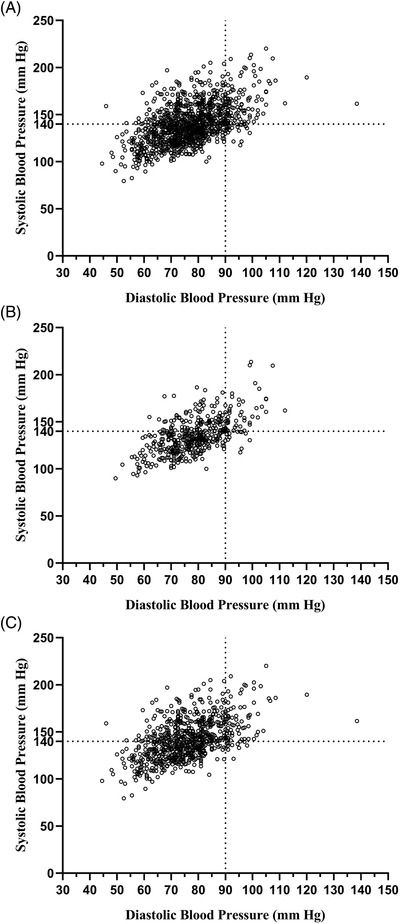
Relationship between systolic blood pressure and diastolic blood pressure in all participants (A), participants <65 years of age (B), and participants ≧65 years (C)

**FIGURE 2:**
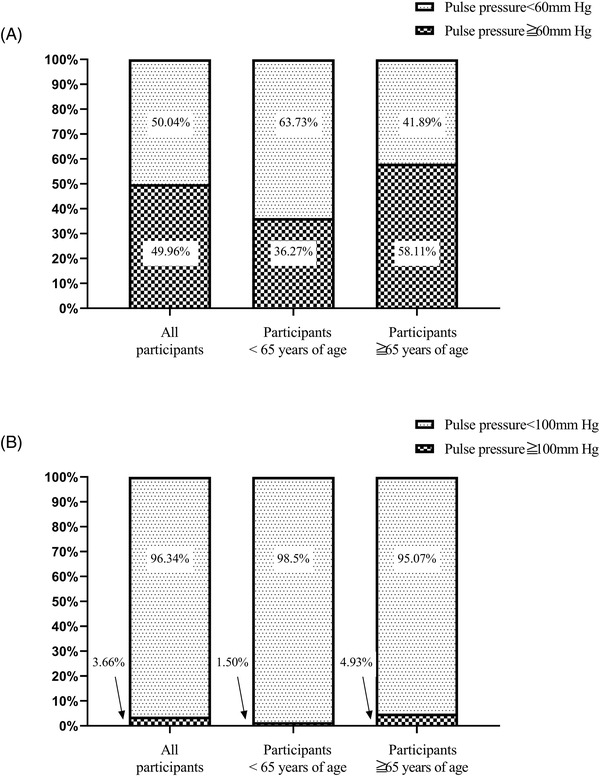
Percentage of wide pulse pressure in all participants, participants <65 years of age, and participants ≧65 years

**Figure** **1** shows the relationship between SBP and DBP measurements in all participants, as well as in those in the two age groups. In all participants, the prevalence rates of a wide PP defined as ≥60 or ≥100 mmHg were 49.96% (628/1257) and 3.66% (46/1257), respectively. When wide PP was defined as ≥60 mmHg, the prevalence of a wide PP was significantly higher in older participants than in younger participants (58.11% vs. 36.27%, *p <* .001). Similar results were obtained when a wide PP was defined as ≥100 mmHg; older participants had a significantly higher prevalence rate than younger participants (4.93% vs. 1.50%, *p* = .002; **Figure** **2**).

### 3.2 Relationship of blood pressure components with cardiovascular events and all‐cause death

In total, 173 cardiovascular events occurred in the 4.84‐year observation period (6,081.8 person‐years), including 58 coronary events (33.5%), 113 stroke events (65.3%), and two peripheral vascular events (1.2%). During the follow‐up period, 155 deaths occurred, and 156 participants (12.41%) were lost to follow‐up. The incident rates of cardiovascular events and mortality of all participants were 27.62 and 24.40 per 1000 person‐years, respectively.

**Table** **2** details the crude and adjusted HRs and 95% CIs of blood pressure for the risks of cardiovascular events and all‐cause death. After adjusting for sex, age, smoking status, alcohol consumption, diabetes mellitus, dyslipidemia, hsCRP, eGFR, and antihypertensive treatment, SBP, PP, and wide PP (either ≥60 or ≥100 mmHg) were significantly associated with the risk of cardiovascular events (all *p* < .05). A wide PP (≥60 mmHg) was significantly associated with a 42% increased risk for cardiovascular events (HR: 1.42, 95% CI: 1.02–1.91). The HR of a wide PP (≥100 mmHg) was 2.39 (95% CI: 1.41–4.08). Similar findings were obtained for all‐cause death except for a wide PP (≥60 mmHg). SBP, PP, and wide PP (≥100 mmHg) were significantly associated with the risk of all‐cause death (all *p* < .05). The fully adjusted HR of a wide PP (≥100 mmHg) was 1.97 (95% CI: 1.40–3.42). Notably, no significant association was observed between DBP and the risk of all‐cause death, and DBP was only associated with a marginally significant 1% increased risk for cardiovascular events (HR: 1.01, 95% CI: 1.00–1.03, *p* = .051).

**TABLE 2:** Cox regression analyses of blood pressure for cardiovascular disease events and all‐cause death

	Cardiovascular disease events		All‐cause death	
	HRs (95% CI)	*p*‐value	HRs (95% CI)	*p*‐value
Systolic blood pressure				
Crude	1.02 (1.01‐1.03)	<.001	1.02 (1.01‐1.02)	<.001
Model 1	1.02 (1.01‐1.02)	<.001	1.01 (1.00‐1.02)	.006
Model 2	1.01 (1.01‐1.02)	<.001	1.01 (1.01‐1.02)	.008
Diastolic blood pressure				
Crude	1.01 (1.00‐1.03)	.040	1.00 (0.98‐1.01)	.777
Model 1	1.02 (1.00‐1.03)	.090	1.01 (0.99‐1.02)	.264
Model 2	1.01 (1.00‐1.03)	.051	1.01 (0.99‐1.02)	.289
Pulse pressure				
Crude	1.02 (1.01‐1.03)	<.001	1.02 (1.02‐1.03)	<.001
Model 1	1.02 (1.01‐1.03)	<.001	1.01 (1.00‐1.02)	.009
Model 2	1.01 (1.01‐1.02)	<.002	1.01 (1.00‐1.02)	.011
Wide pulse pressure (≧60 mmHg)				
Crude	1.73 (1.27‐2.38)	<.001	1.58 (1.14‐2.19)	.006
Model 1	1.51 (1.10‐2.09)	.012	1.11 (0.80‐1.55)	.539
Model 2	1.42 (1.02‐1.91)	.037	1.08 (0.76‐1.52)	.672
Wide pulse pressure (≧100 mmHg)				
Crude	3.61 (2.15‐6.05)	<.001	3.51 (2.06‐5.98)	<.001
Model 1	2.91 (1.72‐4.92)	<.001	2.10 (1.22‐3.62)	.008
Model 2	2.39 (1.41‐4.08)	<.001	1.97 (1.40‐3.42)	.015

Abbreviations: CI, confidence interval; HRs, hazard ratios.

Model 1: adjusted for sex and age.

Model 2: adjusted for variables in model 1 plus smoking status, alcohol consumption, diabetes mellitus, dyslipidemia, high‐sensitivity C‐reactive protein, estimated glomerular filtration rate, and antihypertensive treatment.

**FIGURE 3:**
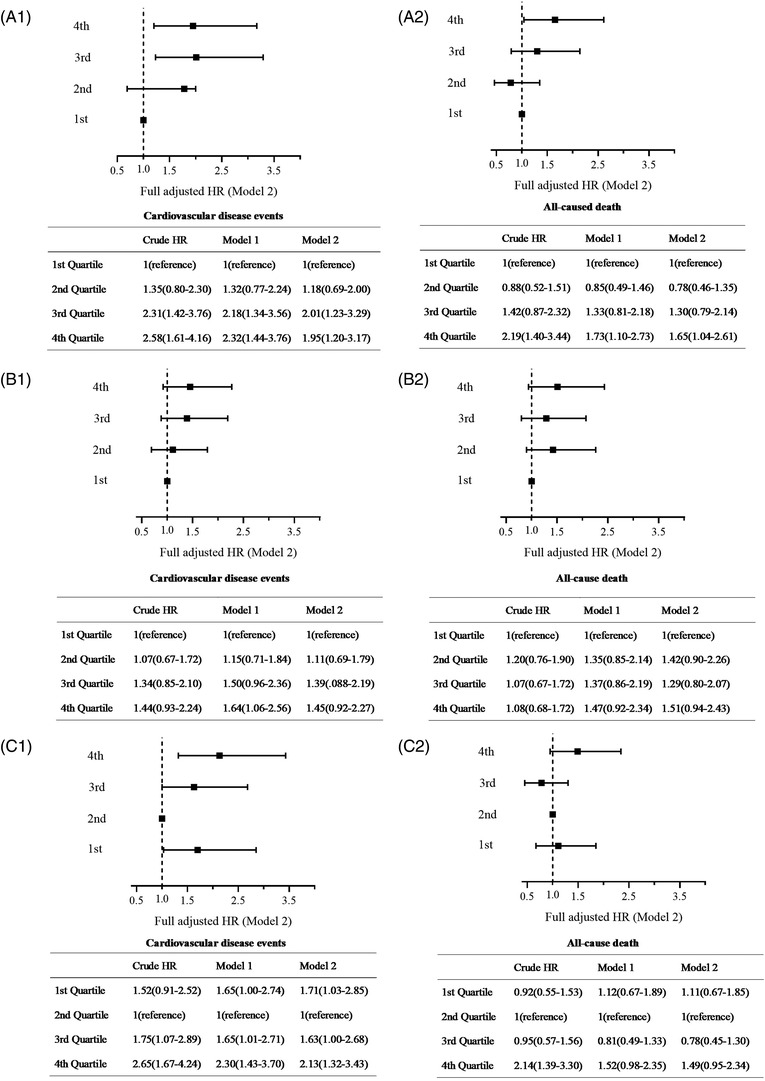
Risks of cardiovascular disease events and all‐cause death by blood pressure Hazard ratio (HR) and 95% confidence interval (CI) using Cox models. Model 1: adjusted for sex and age. Model 2: adjusted for variables in model 1 plus smoking status, alcohol consumption, diabetes mellitus, dyslipidemia, high‐sensitivity C‐reactive protein, estimated glomerular filtration rate, and antihypertensive treatment. (A1) HR and 95% CI for cardiovascular disease events for systolic blood pressure (SBP) subgroups. (1st quartile as the reference); (A2) HR and 95% CI for all‐cause death for SBP subgroups. (1st quartile as the reference); (B1) HR and 95% CI for cardiovascular disease events for diastolic blood pressure (DBP) subgroups. (1st quartile as the reference); (B2) HR and 95% CI for all‐cause death for DBP subgroups. (1st quartile as the reference); (C1) HR and 95% CI for cardiovascular disease events for pulse pressure (PP) subgroups. (2nd quartile as the reference); (C2) HR and 95% CI for all‐cause death for systolic PP subgroups. (2nd quartile as the reference)

When participants were categorized by SBP, DBP, and PP quartiles, the association between PP and cardiovascular events appeared to be J‐shaped. As shown in **Figure** **3**, participants in the highest PP quartile (>72.5 mmHg) had the highest risk of cardiovascular events (fully adjusted HR: 2.13, 95% CI: 1.32–3.43) compared with those in the second PP quartile (reference group). In addition to the increased risk associated with elevated PP, we also observed an increased risk associated with low PP. The lowest PP quartile (<50 mmHg) increased the risk of cardiovascular events by 71% (fully adjusted HR: 1.71, 95% CI: 1.03–2.85) compared with the reference group.

SBP had approximately linear associations with outcomes. Participants in the highest SBP quartile had a higher risk of cardiovascular events (fully adjusted HR: 1.95, 95% CI: 1.20–3.17) and all‐cause death (fully adjusted HR: 1.65, 95% CI: 1.04–2.61) compared with those in the first SBP quartile. No significant association was observed between DBP and outcomes (**Figure** **3**).

### 3.3 Blood pressure and outcomes in the two age groups

The results of Cox regression analyses evaluating the effects of blood pressure parameters on cardiovascular events and all‐cause death by age are shown in **Table** **3**. As shown in **Figure** **2**, the prevalence of a wide PP defined as ≥100 mmHg was low, especially in participants aged <65 years. Considering this finding, we assessed the associations of SBP, DBP, PP, and wide PP (≥60 mmHg) with cardiovascular events and all‐cause death in both age groups. In participants aged <65 years, DBP was significantly associated with a 3% increased risk for cardiovascular events (HR: 1.03, 95% CI: 1.01–1.06) in the fully adjusted model. No significant association was observed for SBP, PP, and wide PP (≥60 mmHg) regarding the risk of cardiovascular events and all‐cause death in participants aged <65 years. In participants aged ≥65 years, SBP and PP were significantly associated with cardiovascular events and all‐cause death in the fully adjusted model (all *p* < .05). Wide PP (≥60 mmHg) was associated with a 69% increased risk of cardiovascular events (HR: 1.69, 95% CI: 1.10–2.25), whereas a significant association with all‐cause death (HR: 1.10, 95% CI: 0.76–1.59) was not observed.

**TABLE 3:** Cox regression analyses of blood pressure for cardiovascular disease events and all‐cause death by age

	Participants <65 years of age (n = 466)				Participants ≧65 years (n = 791)			
	Cardiovascular disease events	*p*‐value	All‐cause death	*p*‐value	Cardiovascular disease events	*p*‐value	All‐cause death	*p*‐value
Systolic blood pressure								
Crude	1.02 (1.00‐1.03)	.023	1.02 (1.00‐1.04)	.085	1.02 (1.01‐1.03)	<.001	1.01 (1.00‐1.02)	.005
Model 1	1.02 (1.00‐1.03)	.021	1.02 (1.00‐1.04)	.107	1.02 (1.01‐1.02)	<.001	1.01 (1.00‐1.02)	.018
Model 2	1.01 (1.00‐1.03)	.105	1.02 (0.99‐1.04)	.234	1.01 (1.01‐1.02)	.001	1.01 (1.01‐1.02)	.020
Diastolic blood pressure								
Crude	1.04 (1.01‐1.07)	.005	1.02 (0.98‐1.07)	.298	1.01 (0.99‐1.02)	.275	1.00 (0.99‐1.02)	.819
Model 1	1.04 (1.01‐1.07)	.004	1.03 (0.98‐1.08)	.214	1.01 (1.00‐1.03)	.196	1.01 (0.99‐1.02)	.446
Model 2	1.03 (1.01‐1.06)	.021	1.03 (0.98‐1.08)	.258	1.00 (0.99‐1.02)	.425	1.00 (0.99‐1.02)	.475
Pulse pressure								
Crude	1.01 (0.99‐1.03)	.327	1.02 (0.99‐1.05)	.149	1.02 (1.01‐1.03)	<.001	1.02 (1.01‐1.03)	.001
Model 1	1.01 (0.99‐1.03)	.355	1.02 (0.99‐1.05)	.243	1.02 (1.01‐1.03)	<.001	1.01 (1.00‐1.02)	.018
Model 2	1.01 (0.99‐1.02)	.633	1.01 (0.98‐1.04)	.456	1.02 (1.01‐1.03)	<.001	1.01 (1.01‐1.02)	.019
Wide pulse pressure (≧60 mmHg)								
Crude	1.29 (0.72‐2.28)	.391	1.23 (0.47‐3.22)	.678	1.80 (1.21‐2.69)	.004	1.24 (0.87‐1.76)	.241
Model 1	1.27 (0.71‐2.26)	.418	1.10 (0.41‐2.91)	.855	1.70 (1.14‐2.54)	.010	1.11 (0.78‐1.59)	.554
Model 2	1.17 (0.65‐2.11)	.594	0.96 (0.34‐2.59)	.939	1.69 (1.12‐2.55)	.012	1.10 (0.76‐1.59)	.622

Abbreviations: CI, confidence interval; HRs, hazard ratios.

Model 1: adjusted for sex and age.

Model 2: adjusted for variables in model 1 plus smoking status, alcohol consumption, diabetes mellitus, dyslipidemia, high‐sensitivity C‐reactive protein, estimated glomerular filtration rate, and antihypertensive treatment.

### 3.4 ROC analysis

**FIGURE 4:**
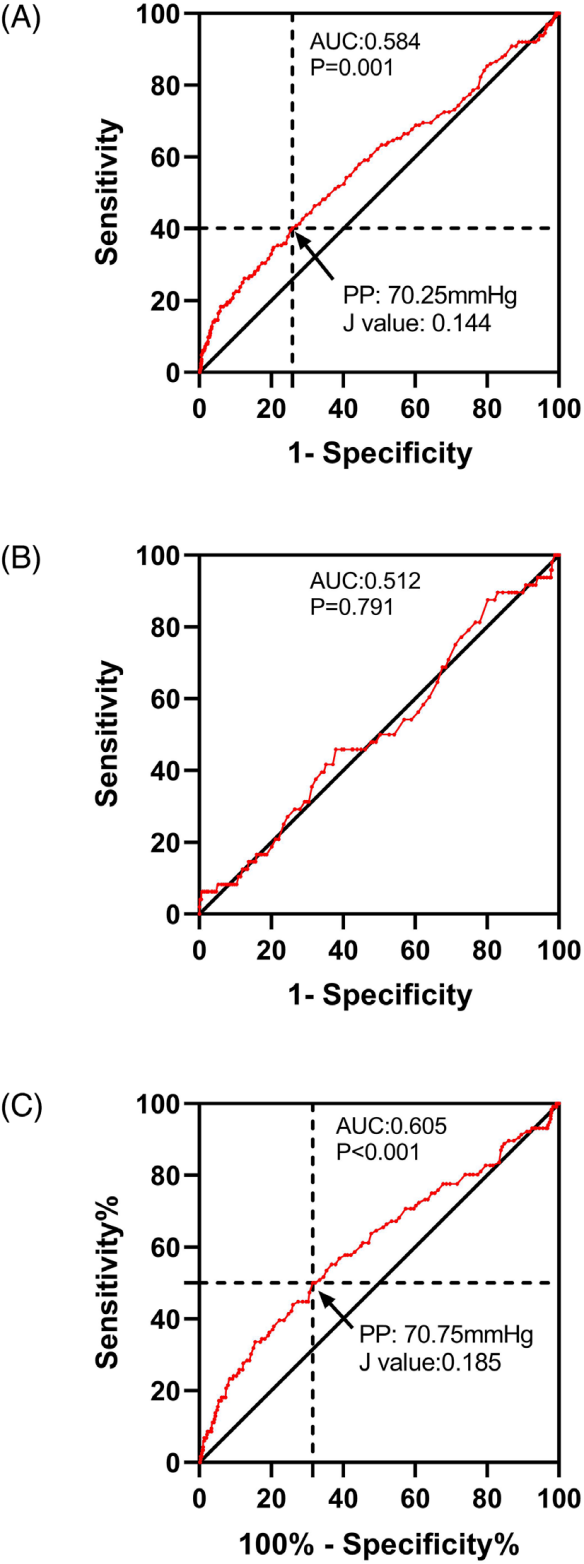
Receiver‐operating characteristic curves for prediction of cardiovascular disease events by pulse pressure (PP) in all participants (A), participants <65 years of age (B), and participants ≧65 years (C) during 4.84‐years observation period. AUC, area under the curve. The ROC analyses revealed a significant AUC in all participants and participants ≧65 years. We calculated the Youden's index. PP = 70.25 mmHg displayed the highest Youden's J value (0.584) in all participants (A), shown in black arrow; PP = 70.75 mmHg displayed the highest Youden's J value (0.584) in participants ≧65 years (C), shown in black arrow

**FIGURE 5:**
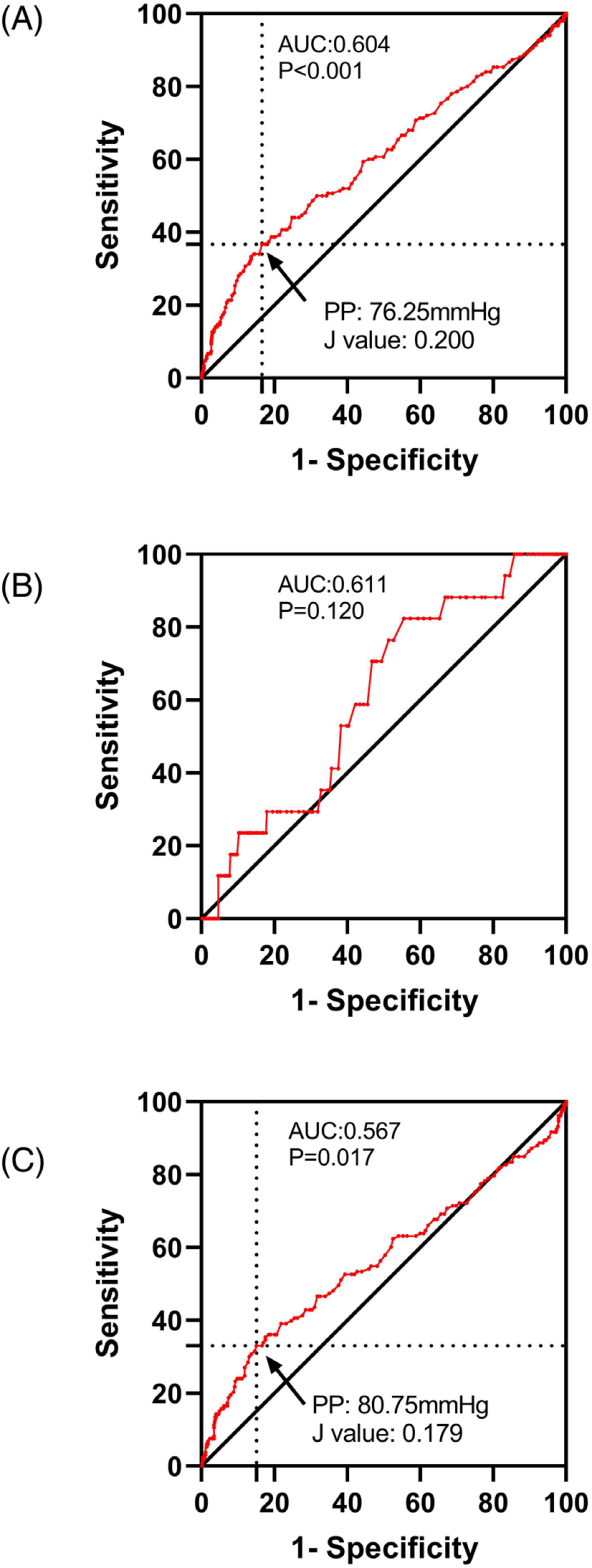
Receiver‐operating characteristic curves for prediction of all‐cause death by pulse pressure (PP) in all participants (A), participants <65 years of age (B), and participants ≧65 years (C) during 4.84‐years observation period. AUC, area under the curve. The ROC analyses revealed a significant AUC in all participants and participants ≧65 years. We calculated the Youden's index. PP = 76.25 mmHg displayed the highest Youden's J value (0.200) in all participants (A), shown in black arrow; PP = 80.75 mmHg displayed the highest Youden's J value (0.179) in participants ≧65 years (C), shown in black arrow

Next, PP levels were analyzed to determine the ROC curves for obtaining optimal cutoff points that predict the risks of cardiovascular events and all‐cause death during the 4.84‐year follow‐up (**Figures** **4** and **5**). The optimal cutoff points of PP for predicting the risk of cardiovascular events were 70.25 mmHg (sensitivity: 40.24%, specificity: 74.11%) and 70.75 mmHg (sensitivity: 50.00%, specificity: 68.50%) in all participants and older participants, respectively. The cutoff points of PP for the prediction of all‐cause death were 76.25 mmHg in all participants (sensitivity: 36.67%, specificity: 83.35%) and 80.75 mmHg in older participants (sensitivity: 33.08%, specificity: 84.85%). In participants aged <65 years, the ROC analyses revealed a non‐significant AUC for cardiovascular events and all‐cause death (AUC: 0.512, *p* = .791 and AUC: 0.611, *p* = .120, respectively; **Table** **4**).

**TABLE 4:** ROC analysis results for the prediction of cardiovascular disease events and all‐cause death by pulse pressure

	AUC (95% CI)	*p*	Cut‐off (mmHg)	Youden index	Sensitivity (%)	Specificity (%)
Cardiovascular disease events						
Overall	0.584 (0.533‐0.635)	.001	70.25	0.144	40.24	74.11
Participants <65 years of age	0.512 (0.424‐0.599)	.791	‐	‐	‐	‐
Participants ≧65 years	0.605 (0.544‐0.665)	<.001	70.75	0.185	50.00	68.50
All‐cause death						
Overall	0.604 (0.551‐0.657)	<.001	76.25	0.200	36.67	83.35
Participants <65 years of age	0.611 (0.492‐0.731)	.120	‐	‐	‐	‐
Participants ≧65 years	0.567 (0.507‐0.627)	.017	80.75	0.179	33.08	84.85

**Abbreviations**: ROC, receiver operating characteristic; AUC, area under the curve

## 4 DISCUSSION

We found that in community‐dwelling individuals without a history of cardiovascular disease, SBP and PP were predictors of cardiovascular events and death, especially in older adults. DBP was considerably less important than SBP and PP as it only independently influenced cardiovascular outcomes in middle‐aged participants. We found a J‐shaped relationship between PP and the incidence of cardiovascular events, and a PP of 50–60 mm Hg was associated with the lowest risk of cardiovascular events. Cutoff points of a wide PP for predicting the risks of cardiovascular events and all‐cause death were 70.25 and 76.25 mmHg, respectively.

Previous studies prioritized SBP over DBP as a predictor of cardiovascular events.^5^, ^6^ Moreover, additional studies reported that DBP was not independently associated with cardiovascular events,^29^ cardiovascular mortality,^10^ symptomatic peripheral artery disease,^30^ and target organ damage^31^ in individuals regardless of age. Consequently, slogans, such as “Systolic pressure is all that matters” and “Abandoning diastole” made the headlines.^6^, ^11^ Current cardiovascular risk estimation tools^13^ and hypertension guidelines^14^ assign more importance to SBP and less to DBP. More recently, the Systolic Blood Pressure Intervention Trial trial^3^ further promoted the benefits of intensive SBP control in high‐risk cardiovascular patients and the benefit of the intensive SBP lowering did not differ by baseline DBP.

Our results show that although SBP is indeed a dominant predictor, DBP should not be ignored in middle‐aged individuals. In line with our results, the Framingham Heart Study demonstrated in a cohort of 6539 participants between 20 and 79 years of age who were free of coronary heart disease, that DBP was the strongest predictor of coronary heart disease risk (HR per 10 mmHg increment: 1.34, 95% CI: 1.18–1.51) rather than SBP (HR: 1.14, 95% CI: 1.06–1.24) in the group <50 years of age. With advancing age, a gradual shift from DBP to SBP and PP as predictors of coronary heart disease was observed.^19^ Similarly, the MOnica, Risk, Genetics, Archiving, and Monograph Project, a cohort study with 68,551 participants without cardiovascular disease, showed that the HR for DBP decreased with age (p < .001) and was not influenced by other cardiovascular risk factors.^20^ Isolated diastolic hypertension constitutes the most frequent phenotype in hypertensive individuals aged <50 years.^32^ Age‐dependent development of arterial stiffening results in a higher SBP in older individuals.^19^ Compared to older individuals, younger people have a low cardiovascular risk. However, we still observed that DBP increased the risk of cardiovascular events by 3% in the middle‐aged group. These findings from the current and previous studies imply that age‐related shifts in both SBP and DBP should be considered cardiovascular risk factors, and primary preventive measures may be more effective if applied earlier during the progression of hypertension.^19^

Our findings do not confirm the results of previous studies, which described a J‐curve phenomenon^8^, ^9^ between DBP and cardiovascular risk. A J‐curve, that is, an increase in cardiovascular risk below or above a certain DBP, has been described in patients with coronary artery disease^8^, ^9^ and in high‐risk populations.^33^ Additionally, a DBP of <60 mmHg was associated with incident coronary heart disease, but not with stroke, in the Atherosclerosis Risk in Communities (ARIC) cohort.^34^ Importantly, these studies were conducted in high‐risk cardiovascular populations and did not find a J‐shaped relationship between DBP and stroke. Notably, our community cohort had no cardiovascular disease history and had a higher incidence of stroke than coronary event, consistent with the findings of a previous report.^15^ A direct J‐curve of DBP may be of greater importance in patients with limitations to coronary perfusion^8^ or in those with conditions involving end‐organ microcirculatory abnormalities.^5^ As also demonstrated in the ARIC cohort, low DBP levels might harm the myocardium and are associated with subsequent coronary artery disease.^34^

PP, an indicator of diffuse vascular stiffening and vascular aging, has been reported to be independently associated with adverse cardiovascular outcomes and mortality in patients with hypertension^24^ and coronary artery disease,^9^ patients with heart failure with reduced^35^ and preserved ejection fraction,^36^ and patients with atherothrombosis.^37^ High PP and low PP states reflect adverse hemodynamic conditions and poor perfusion states,^37^ respectively. In line with our results, J‐shaped patterns for the link between PP and cardiovascular events have been shown in the Prospective Observational Longitudinal Registry of Patients with Stable Coronary Artery Disease registry^9^ and Reduction of Atherothrombosis for Continued Health (REACH) registry.^37^ The REACH study also reported that PP is a particularly useful indicator in older patients because SBP and DBP tend to diverge with aging.

Recently, clinical trials^33^ and hypertension guidelines^7^, ^14^ have focused more on SBP and DBP than PP control. Considering that studies identified PP as a risk factor for cardiovascular events and death, PP management may serve as a therapeutic target in hypertension. Short‐term dietary supplementation with folic acid reduced PP and increased systemic arterial compliance.^38^ These responses were independent of homocysteine or folate concentrations. The Prospective Comparison of Angiotensin Receptor Neprilysin Inhibitor with Angiotensin Receptor Blockers Global Outcomes trial showed that PP was predictive of adverse events in patients with heart failure with preserved ejection, and sacubitril/valsartan may have beneficial effects through PP reduction.^36^

However, the definition of a “wide PP” to predict cardiovascular risk is controversially discussed. Current hypertension guidelines do not include a target PP.^7^, ^14^, ^15^ Various studies showed that a wide PP, which is defined as a value of >50,^16^ >60,^17^, ^24^ >65,^18^ >70,^37^ or >80 mmHg^39^ is associated with cardiovascular risk and organ damage. The current study determined the cutoff points of a wide PP for predicting the risks of cardiovascular events and all‐cause death as 70.25 and 76.25 mmHg, respectively. Differences in cutoff points defining a wide PP among these studies may be due to differences in population characteristics and ethnic backgrounds.

Our results should be interpreted in the context of certain limitations. First, due to the relatively small sample size, we cannot separately analyze the association of blood pressure components with the risks of coronary events and stroke events. Second, selection bias existed because 1010 participants (43.84%) were excluded for refusing to provide blood samples, and 37 participants (1.61%) were excluded for missing important information. We compared the characteristics of participants with or without laboratory assessments/important information from a total of 2304 participants without a history of cardiovascular disease. The group of participants who completed laboratory assessments or had important information included younger individuals (69.16 ± 8.10 vs. 73.66 ± 9.33 years, *p* < .001) and had a lower proportion of male individuals (43.76% vs. 48.52%, *p* = .022) and higher proportion of drinkers (30.71% vs. 24.17%, *p* < .001) than the group of participants who did not complete the laboratory assessments or had missing information. Thus, the representativeness of the population could thus be weaker than expected. Third, we did not have data for blood pressure levels, whole antihypertensive medication, and newly identified risk factors during the follow‐up period. Although potential confounding risk factors, including antihypertensive therapy at baseline survey, were adjusted for as covariables in the multivariate Cox regression analysis, the absence of important data such as whole antihypertensive medication and newly identified risk factors during the follow‐up period still has an impact on the effects of blood pressure on cardiovascular outcomes and death. However, the findings were obtained under real‐world conditions and may have greater external validity than those in highly selected populations of other studies.^24^ Fourth, given that middle‐aged participants are at a lower risk of cardiovascular events than older participants, a longer follow‐up time will be needed in future studies. Fifth, participants with severe aortic regurgitation or stenosis were not excluded from the study as echocardiographic examinations were not performed, which may have altered the PP levels in the study population. Sixth, our results in a Chinese community‐dwelling population without a history of cardiovascular disease may not be applicable to populations with other ethnic or socioeconomic characteristics.

Nevertheless, our study also has certain strengths. We performed a prospective cohort study to explore the association between blood pressure components and adverse outcomes. This study was under strict quality control, and potential confounding risk factors were determined and adjusted for as covariables in the multivariate Cox regression analysis. Oscillometric devices were used in this study to obtain valid blood pressure measurements, which may have reduced human errors associated with auscultatory measurements.^40^

## 5 CONCLUSIONS

In conclusion, this prospective cohort study shows that SBP and PP are associated with cardiovascular events and death in community‐dwelling populations aged ≥55 years. DBP independently influences the risk of cardiovascular events in middle‐aged populations. Cardiovascular events and all‐cause death are associated with high, but also low, PP levels, and the data of this study suggest cutoff values of wide PP (>70.25 and >76.25 mmHg, respectively). The results indicate that age‐related shifts in both SBP and DBP should be considered cardiovascular risk factors, and PP may serve as a therapeutic target in hypertension. Caution should be exercised in avoiding excessively high or low PP values when using antihypertensive medication.

## CONFLICT OF INTEREST

The authors have no competing interests to declare.

## AUTHOR CONTRIBUTIONS

Zhongying Zhang, Zhe Tang, and Xianghua Fang contributed to the study concept and design. Zhongying Zhang, Xiang Gu, Shaochen Guan, Hongjun Liu, and Yan Zhao contributed to data collection. Zhongying Zhang and Xiaoguang Wu analyzed and interpreted the data. Zhongying Zhang drafted and revised the manuscript. All authors reviewed and approved the final manuscript.

## ETHICS APPROVAL STATEMENT

The Ethics Committee of Xuanwu Hospital, Capital Medical University, Beijing, China, approved this study (No.2018‐038). This study was performed in accordance with the Declaration of Helsinki.

## PATIENT CONSENT STATEMENT

All participants provided written informed consent.

## PERMISSION TO REPRODUCE MATERIAL FROM OTHER SOURCES

The paper does not reproduce material from other sources.
